# Research on Named Entity Recognition Method of Metro On-Board Equipment Based on Multiheaded Self-Attention Mechanism and CNN-BiLSTM-CRF

**DOI:** 10.1155/2022/6374988

**Published:** 2022-07-06

**Authors:** Junting Lin, Endong Liu

**Affiliations:** School of Automation & Electrical Engineering, Lanzhou Jiaotong University, Lanzhou 730000, China

## Abstract

Massive and complex unstructured fault text data will be generated during the operation of subway trains. A named entity recognition model of subway on-board equipment based on Multiheaded Self-attention mechanism and CNN-BiLSTM-CRF is proposed to address the issue of low recognition accuracy and incomplete recognition features of unstructured fault data named entity recognition task of subway on-board equipment: BiLSTM-CNN parallel network extracts context feature information and local attention information, respectively; In the MHA layer, the features learned from different dimensions are fused through the Multiheaded Self-attention mechanism, and the dependencies of various ranges in the sequence are captured to yield the internal structure information of the features. The conditional random field CRF is used to learn the internal relationship between tags to ensure their sequence. This model is tested with other named entity recognition models on the marked subway on-board fault data. The experimental results demonstrate that this model is able to recognize 10 kinds of labels in the dataset. Moreover, the recognition effect of each label has a good performance in the three evaluation indexes of *P*, *R*, and *F*1 score. Moreover, the weighted average evaluation indexes Avg − *P*, Avg − *R*, and Avg − *F*_1_ of 10 labels in this model reach the highest 95.39%, 95.48%, and 95.37%, which has high evaluation indexes and can be applied to the named entity recognition of Metro on-board equipment.

## 1. Introduction

Subway on-board equipment is the basic piece of equipment guaranteeing the safe operation of the subway train. On-board equipment is also constantly upgraded owing to the rapid development of China's urban rail transit. With the accumulation of subway operation mileage and operation time, a consequential amount of fault data about on-board equipment has been generated. These data record the detailed fault information in the form of text, containing useful knowledge of fault diagnosis and processing. However, given that it is stored in the form of unstructured text, it is not conducive for computer processing and understanding. It has long been delved into by field engineers and technicians who suggest that the fault knowledge cannot be reused efficiently. Therefore, for these large amounts of unstructured subway fault knowledge, knowledge entities should be efficiently identified and integrated, the fault cases and treatment methods in the fault knowledge should be identified, the subway knowledge map should be built, and field personnel should be provided with accurate subway fault information. The human-computer interaction platform provides field personnel with three kinds of information: subway fault information, fault causes, and other knowledge information. Moreover, the named entity recognition task related to subway on-board equipment also establishes a knowledge base to serve subway fault diagnosis, subway train information service, and subway information intelligent recommendation [1].

Named entity recognition (NER) [2] is an essential component of natural language processing (NLP) [3]. It aims to identify various named entities from the original text, such as name, location, and organization. It can subsequently extract the concerned information in the fault text data as named entities [4]. The extracted entities can subsequently pave the way for other NLP tasks. The methods of named entity recognition mainly include rule-based methods, statistics-based methods, and deep learning-based methods: Pan [5, 6] constructed the rule base of named entity recognition and used the method of rule matching to identify named entities. However, the rule writing based on rules and dictionary methods requires the involvement of domain experts, thereby requiring high language knowledge and poor portability. Therefore, statistical machine learning was employed to deal with the NER problem. In statistical machine learning, the main algorithms suitable for sequence annotation tasks are: Hidden Markov models (HMM) [7, 8], Maximum Entropy Markov models (MEMM) [9], conditional random field (CRF) [10], etc. However, the method based on machine learning requires a substantial amount of labeled data to train the model, requiring significant manpower, thereby leaving much room for improvement in recognition accuracy. In recent years, deep neural network has been used to realize the key tasks in the knowledge map due to the advent of deep learning technology [11], garnering extensive attention. The use of named entity recognition technology to identify entities in subway operation and maintenance logs is a basic step in the conversion of subway fault text into structured data, thereby laying a foundation for mining and developing the rich knowledge contained in a large amount of fault data recorded during subway operation [12]. The current mainstream deep learning solutions tend to embed layer and Bidirectional long short-term memory (BiLSTM) layer, allowing the machine to directly learn the features. It subsequently directly inputs the learned features into CRF, thereby circumventing the tedious task of manually formulating the feature function [13]. Literature [14, 15] uses the neural network model to learn the internal representation of text on a large number of unmarked datasets, which does not require the setting of artificial features. Literature [16] adopts the long short-term memory (LSTM) neural network model, boosting the performance of word segmentation. However, this method cannot yield the semantic information behind the sentence. Literature [17, 18] proposes that CRF is used as the processing mode of output processing layer on the basis of bidirectional LSTM, effectively improving the performance of the model. Furthermore, convolutional neural network (CNN) [19] has also achieved desirable results in solving NER problems; literature [20] uses CNN to obtain multilevel features, thereby yielding local attention information and improving the sensitivity of entity boundary information; literature [21] adopts the serial strategy of CNN and LSTM-CRF to recognize the named entity of the conll2003 English dataset, and obtains a higher F1 value. However, LSTM network cannot capture text information in both directions. Document [22] uses the Bidirectional gate recurrent unit (BiGRU) and CRF combined with CNN for named entity recognition, and uses the connection vector including affix vector, part of speech vector, and word vector as input. It ultimately outputs through the CRF layer, which can address the issue of automatic named entity recognition and exert a desirable effect on entity recognition. Document [18] proposed a method to fuse character and word vectors. It adopted the Chinese named entity recognition method of BiLSTM-CRF to effectively extract two features at character and word level, thereby effectively improving the accuracy of named entity recognition. Literature [23] adopts the BERT-CRF model, extracts the global features of the input sequence through the Bert pretraining model, adds the CRF layer at the end of the model, introduces hard constraints, and constructs the model framework of named entity recognition. However, the Bert model has a lengthy pretraining time, and it is only used as a transfer learning model, which is hindered by insufficient information recognition ability for small areas. Much research has been conducted in the field of railway text data analysis. In terms of named entity recognition, Yang [24] used word2vec to represent the characteristics of railway accident faults, and used BiLSTM-CRF to realize the named entity recognition of railway electrical service accident faults. Literature [17] uses BiLSTM-CRF to realize the named entity recognition of high-speed railway signal equipment and puts forward the entity relationship representation method of multidimensional word segmentation features, thereby achieving high evaluation indexes for the task of named entity recognition of high-speed railway signal equipment.

Based on the above literature research, this paper proposes a named entity recognition method for Metro on-board equipment based on multiheaded self-attention (MHA) and BiLSTM-CNN- CRF. The core idea of the method is as follows:

1. YMDAA is used to complete the sequence annotation [25], and the location, phenomenon, and measure of the fault in the subway on-board fault text are marked and exported in an Ann format file. The file is subsequently read through Python and added to the BMEO label to complete the preannotation of fault text data.
2. The tag and word sequence of the prelabeled fault text are input into the word2vec model and transformed into feature vectors. The strategy of CNN and BiLSTM working in parallel is adopted, whereby CNN and BiLSTM work simultaneously, extract the context and local attention features in the fault text, respectively, and ultimately fuse the two kinds of information.
3. The multihead self-attention mechanism is adopted to give higher weight to the more important information in the input word sequence and label sequence. This mechanism can boost the sensitivity of the machine towards important information, mining the association between different input features to extract the feature vector containing other word information. The recognition ability of the machine to feature information can be more comprehensive by defining the number of heads of multiple groups of attention mechanisms, extracting important features from different dimensions, and splicing and linear processing these features [26].

## 2. Design of Named Entity Recognition Model Based on Multihead Self-Attention Mechanism and BiLSTM-CNN-CRF

**Figure 1:**
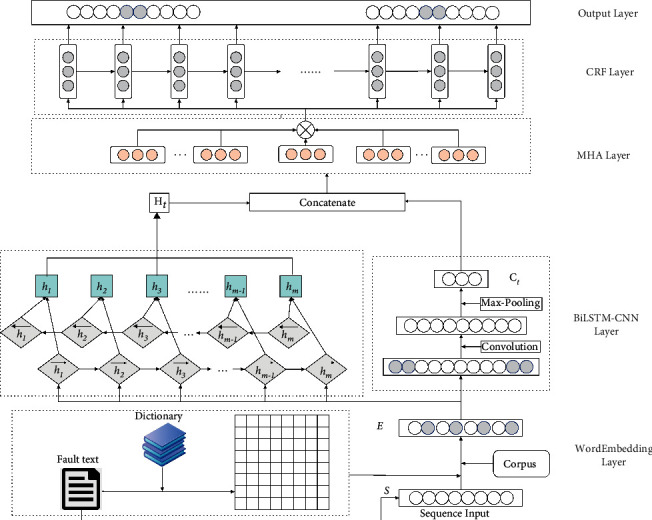
The overall model architecture of NER.

Based on the multiheaded self-attention mechanism and BiLSTM-CNN-CRF, the named entity recognition model architecture of Metro on-board equipment is illustrated in Figure 1. It includes four main layers: word embedding layer, BiLSTM-CNN layer, MHA layer, and CRF layer.

In the word embedding layer, the subway on-board fault database is first loaded, the fault text records in the database are marked with BMEO through YMMDA, the word vector of large-scale marked text is subsequently trained in the same field as word2vec. The generated word vector is then input into the BiLSTM module and CNN module in the BiLSTM-CNN layer, respectively. The BiLSTM module is used to learn the time characteristics and context information of the text sequence, The CNN module is used to extract the local features in the text. The outputs of BiLSTM and CNN are then spliced and fed to the MHA layer to yield the global features of the text sequence and the correlation strength between words. Finally, the CRF layer marks the output sequence from the MHA layer according to the importance of the features and outputs the entity prediction label.

### 2.1. Word Embedding Layer

Data preprocessing is first performed on the prelabeled subway on-board fault text, which is subsequently segmented. Stop words and low-frequency words are then discarded. The accuracy of word segmentation exerts a direct impact on the training effect of the model, while the Jieba word segmentation tool may fail to identify some proper nouns in this field. Therefore, a dictionary of proper nouns in the subway on-board field should be defined according to relevant data and existing knowledge, to improve the reliability of word segmentation task and lay a foundation for the vectorization of text [27].

In this paper, word2vec model is used to train word vectors, transforming large-scale subway vehicle fault text and label data into low dimensional and dense word vectors. This model can reflect the relationship between words but does not necessarily ensure sufficient training of proper nouns. To address this issue, word2vec is used to train word vectors on the training set data and other corpora in the field. Word2vec trains the word vector through the skip gram model, whereby the central word predicts the words around it and solves the context word vector through the conditional probability value of the intermediate word vector, to fully learn the semantic vector representation [28]. Suppose the sample S is composed of *n* sentences, input the text sequence *S*=[*s*_1_, *s*_2_, ⋯, *s*_*n*_], the *i* sentence in the text sequence is represented as *s*_*i*_=[*w*_*i*1_, *w*_*i*2_, ⋯, *w*_*ik*_], whereby *k* represents the number of words contained in the sentence *s*_*i*_, and w_ik_ represents the *k* word in the *i* sentence. The skip gram model converts the input text sequence into word vector, and further generates the corresponding word vector matrix. **W**_*ij*_ represents the *j* word vector in the *i* sentence, and the word vector matrix of the sentence *s*_*i*_ with the length of *k* is represented as **W**_*i*1:*im*_=[**W**_*i*1_, **W**_*i*2_, **W**_*i*3_, ⋯, **W**_*ik*_]. Finally, **E**=[**W**_11:2*k*_, **W**_21:2*k*_, **W**_31:3*k*_, ⋯, **W**_*n*1:*nk*_], the word vector matrix spliced by *n* sentences in the sample *S*, is used as the output of the word embedding layer.

### 2.2. BiLSTM-CNN Layer

This layer adopts the strategy of BiLSTM and CNN working in parallel, whereby the feature vectors generated by word2vec are input to BiLSTM and CNN networks, respectively; the context features and local attention features are extracted respectively; and the two fusion features are subsequently input to the MHA layer.

#### 2.2.1. BiLSTM

**Figure 2:**
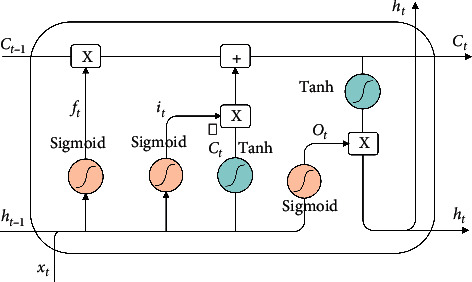
The structure of LSTM.

LSTM effectively calculates and controls the input and output of information by designing gating units in neurons. The design of this gating unit addresses the problem of text sequence length dependence. Its structure is illustrated in Figure 2.

The information of cell state *C*_*t*−1_ is transmitted through the top straight line. The hidden layer state *h*_*t*_ and input *x*_*t*_ at *t* time will modify *C*_*t*_ appropriately and then output to the next time. Moreover, *C*_*t*−1_ will participate in the calculation of *h*_*t*_ output at *t* time, and alter the cell state through the gate structure of LSTM. After connecting *h*_*t*−1_ and *x*_*t*_, calculate with different weight matrices (**W**_*f*_, **W**_*i*_, **W**_*i*_) and offset (*b*_*f*_, *b*_*i*_, *b*_*o*_) through the sigmoid function, and output *f*_*t*_, *i*_*t*_, and *o*_*t*_ respectively. The calculation formula is shown in (1)–(3). The amount of information needed to be forgotten from the previous hidden layer *h*_*t*−1_ is controlled by multiplying *f*_*t*_ and *C*_*t*−1_; the content is planned to (−1, 1) through the function, so that the updated cell $\tilde{C_{t}}$ is multiplied with *i*_*t*_ to control which information needs to be retained. The calculation is shown in formula (4), where **W**_*C*_ denotes the weight matrix. When the information in the cell state *C*_*t*_ is completely updated, as shown in formula (5), it is scaled by tanh and multiplied by *o*_*t*_ to output *h*_*t*_ as the next LSTM hidden layer state. The calculation is shown in formula (6).

(1) $$\begin{array}{c} f_{t}=\sigma \left({\text{W}}_{f}\cdot \left[h_{t-1},x_{t}\right]+b_{f}\right), \end{array}$$

(2) $$\begin{array}{c} i_{t}=\sigma \left({\text{W}}_{i}\cdot \left[h_{t-1},x_{t}\right]+b_{i}\right), \end{array}$$

(3) $$\begin{array}{c} o_{t}=\sigma \left({\text{W}}_{o}\cdot \left[h_{t-1},x_{t}\right]+b_{o}\right), \end{array}$$

(4) $$\begin{array}{c} \tilde{C_{t}}=\tanh\left({\text{W}}_{C}\cdot \left[h_{t-1},x_{t}\right]+b_{C}\right), \end{array}$$

(5) $$\begin{array}{c} C_{t}=f_{t}*C_{t-1}+i_{t}*\tilde{C_{t}}, \end{array}$$

(6) $$\begin{array}{c} h_{t}=o_{t}*\;\tanh\left(C_{t}\right). \end{array}$$

However, given that the unidirectional LSTM model can only capture the information before the sequence and cannot capture the context semantics, Li et al. [27] improved the RNN model to yield the LSTM, which can solve the problems of gradient disappearance and gradient explosion that could occur in the process of long sequence training. BiLSTM is composed of forward propagating LSTM and back propagating LSTM. It captures the above and below information of the current text, respectively, and then combines the feature information extracted from the two directions to yield the text features of remembering the past and the future. The word vector matrix E obtained through the word embedding layer is input to the BiLSTM part in the BiLSTM CNN layer as illustrated in Figure 1. The LSTM forward propagation generates the forward hidden layer state sequence: $H_{1}=\left[\overset{⟶}{h_{1}},\overset{⟶}{h_{2}},\ldots ,\overset{⟶}{h_{m}}\right]$, and the reverse hidden layer state sequence: $H_{2}=\left[\overset{\leftarrow }{h_{1}},\overset{\leftarrow }{h_{2}},\ldots ,\overset{\leftarrow }{h_{m}}\right]$. The forward hidden layer state sequence H_1_ is spliced with the reverse hidden layer state sequence H_2_ to obtain the complete hidden layer state sequence $H_{t}=\left[\overset{⟶}{h_{m}},\overset{\leftarrow }{h_{m}}\right]$, where *m* represents the dimension of the BiLSTM input word vector. This combination of forward and reverse states gives full play to the advantages of BiLSTM and addresses the issue whereby the traditional one-way LSTM model fails to capture the context information. It fully combines the context and extracts the features through the overall environment, which can substantially mitigate feature loss. The hidden layer state sequence **H**_*t*_=[*h*_1_, *h*_2_,…, *h*_*m*_] is the final output of the BiLSTM layer and is input to the MHA layer.

#### 2.2.2. CNN

**Figure 3:**
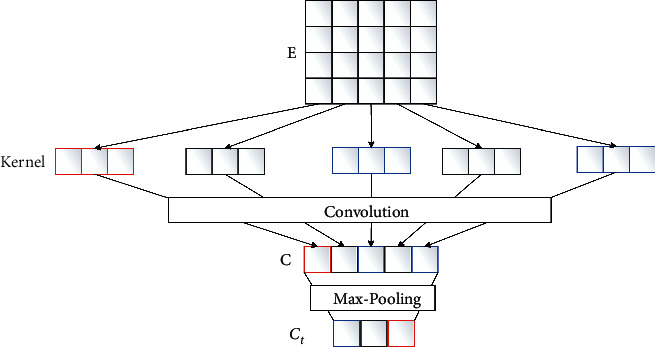
Working principle of the CNN layer.

The word vector matrix set generated by the word embedding layer is input to the CNN layer. The CNN layer includes two steps: convolution and max pooling. Its working process is depicted in Figure 3. Convolution is the use of different sizes of convolution to check the input eigenvector matrix for feature calculation, and then extracting the local feature information of the text. The operation process of convolution can be expressed as follows:

(7) $$\begin{array}{c} c_{i}=f\left(F\cdot W_{\text{i:j}}+b\right), \end{array}$$

where *c*_*i*_ denotes the *i*th eigenvalue of the text output through convolution operation; F represents the matrix corresponding to the convolution kernel; *f* is a nonlinear activation function; · indicates that the two matrices are multiplied by points; **W**_*i*:*j*_ represents the word vector matrix from *i* word to *j* word; and *b* is the offset term. Convolution operation on the characteristic matrix of each word vector is carried out in the input, and the characteristic graph **c** is calculated through formula (8).

(8) $$\begin{array}{c} c=\left(c_{1},c_{2},\cdots ,c_{n}\right). \end{array}$$

The pooling layer samples the text features by setting a fixed step stripe. In this paper, the maximum pooling strategy max pooling is used for pooling processing. This process aims to effectively extract the local key information in the sequence, compress the input feature map, reduce the size of the feature map **c**, to simplify the network calculation, and finally calculate the output fixed length vector **C**_t_ through formula (9).

(9) $$\begin{array}{c} C_{t}=\text{Max}-\text{pooling}\left(c\right). \end{array}$$

### 2.3. MHA Layer

**Figure 4:**
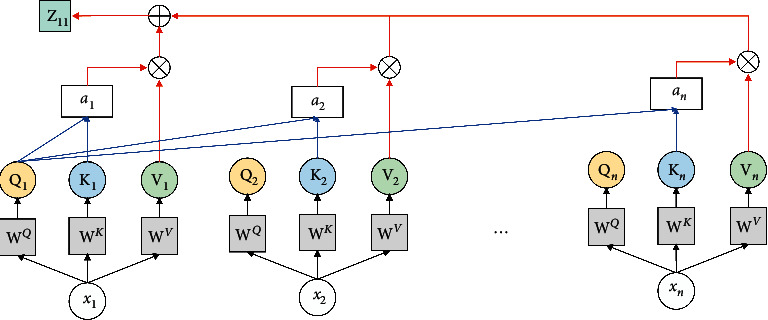
Calculation schematic diagram of self-attention mechanism.

The output **H**_*t*_ of BiLSTM network and the output **C**_*t*_ of CNN network are spliced into a feature vector **X**_*t*_ with a dimension of 320 (the dimension of feature vector **H**_*t*_ is 256 and the dimension of feature vector **C**_*t*_ is 64). However, this feature vector cannot display the importance of key information in the context, which could entail the loss of important information in the named entity recognition task. Therefore, the introduction of the multiheaded self-attention mechanism is essential to learn the dependence between any two words in the sentence, obtain the internal structure information, and distinguish the significance of each word. The calculation principle of self-attention mechanism is illustrated in Figure 4.

Taking the feature *x*_1_ in **X**_*t*_ as an instance, the self-attention mechanism initializes the **W**^*Q*^, **W**^*K*^, and **W**^*V*^ matrices, and obtains **Q**_*n*_, **K**_*n*_, and **V**_*n*_ matrices, respectively, by multiplying with the input feature *x*_1_ points, as shown in formula (10). It then calculates the attention *a*_1*n*_ from formula (12) to represent the correlation degree between the feature *x*_1_ and the feature *x*_*n*_.

(10) $$\begin{array}{c} x_{1}\cdot W^{Q}=Q_{n}x_{1}\cdot W^{K}=K_{n}x_{1}\cdot W^{V}=V_{n}, \end{array}$$

(11) $$\begin{array}{c} a_{1n}=\text{softmax}\left(\frac{Q_{1}K_{n}^{T}}{\sqrt{d_{k}}}\right). \end{array}$$

Among them, **Q**_*n*_ represents the query matrix, **K**_*n*_ represents the key value matrix, **V**_*n*_ represents the score matrix, and *n* is the serial number corresponding to other input features. Through the combination of *a*_1*n*_ and **V**_*n*_, the association **head**_1*n*_ between *x*_1_ and other different features is obtained. The calculation is shown in formula (12). All the feature vectors **head**_1*n*_ are added and the Z_11_ vector is calculated and can represent the connection between the first word and other words through formula (12).

(12) $$\begin{array}{c} head_{1n}=a_{1n}V_{n}, \end{array}$$

(13) $$\begin{array}{c} Z_{11}=Concat\left(head_{11},head_{12},\ldots ,head_{1n}\right). \end{array}$$

**Figure 5:**
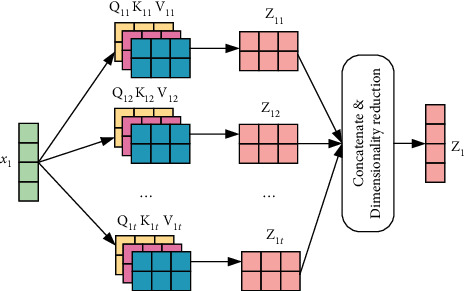
MHA calculation schematic diagram.

The MHA calculation principle is depicted in Figure 5, whereby the multiple groups of **W**^*Q*^, **W**^*K*^, and **W**^*V*^ matrices are initialized, multiple groups of **Q**, **K**, and **V** characteristic matrices are generated through point multiplication, thereby yielding multiple groups of Z_1*t*_. After completing the splicing of multiple groups of Z_1*t*_, the dimension is reduced through linear transformation, whereby *t* denotes the number of self-attention heads, to obtain **Z**_1_ containing other feature information. MHA linearly maps the input features to different information subspaces through different weight matrices, and calculates the same attention function in each subspace, thereby expanding the ability of the model to consider different positions, to fully understand the structure and semantics of sentences. The output *x*_1_′ of the final MHA is calculated by the tanh function from **Z**_1_ and the input characteristic *x*_1_, as shown in formula (14). The MHA value of other features *x*_*n*_ in X_*t*_ is calculated as above.

(14) $$\begin{array}{c} Z_{1}=\tanh\left(x_{1}⊕Z_{1}\right). \end{array}$$

### 2.4. CRF Layer

**Figure 6:**
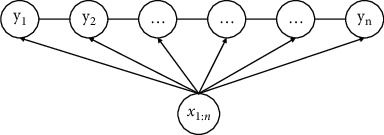
Conceptual diagram of CRF.

BiLSTM only considers the long-term dependency information of sentences but overlooks the dependency between tags. For instance, in the entity tags defined in this paper, b-phenomenon cannot appear after the m-phenomenon. Therefore, CRF needs to be introduced to learn the internal relationship between tags to ensure the sequence of tags. The conceptual diagram of CRF conditional random field is depicted in Figure 6.

The conditional random field model, CRF, is based on the calculation of a given random variable sequence **X**=(*x*_1_, *x*_2_, ⋯, *x*_*n*_). The conditional probability distribution of the random variable sequence **Y**=(*y*_1_, *y*_2_, ⋯, *y*_*n*_) is *P*(**X***| ***Y**), and *n* denotes the sequence length. The model assumes that the random variable sequence satisfies the Markov property:

(15) $$\begin{array}{c} P\left(y_{i}|X,y_{i},\cdots ,y_{n}\right)=P\left(y_{i}|X,y_{i-1},y_{i+1}\right). \end{array}$$

*P*(**X***| ***Y**) can subsequently represent the linear chain conditional random field. In the labeling problem, **X** represents the input observation sequence, **Y** represents the corresponding output mark sequence or state sequence, and the evaluation score Score(**X**, **Y**) can be obtained through formula (16).

(16) $$\begin{array}{c} \text{Score}\left(X,Y\right)=\sum_{i=1}^{n}P_{i,y_{i}}+\sum_{i=0}^{n}W_{y_{i},y_{i+1}}, \end{array}$$

whereby **W** represents the transition matrix, and **W**_*i*,*j*_ represents the state transition score from the *i* character to the *j* character. **P** denotes the weight matrix output by the decoding layer, **P**_*i*,*y*_*i*__ represents the probability that the *i*th word is marked as *y*_*i*_, and exp represents the exponential function of the natural constant *e*. Assuming that the input sentence feature is **X**, the probability distribution of the output sequence *y*′ is *P*(*y*′*| ***X**). Finally, the maximum probability is yielded by the maximum likelihood estimation in the process of fitting the model. The calculation process is shown in formula (17).

(17) $$\begin{array}{c} \log\;P\left(y{}^{\prime }|X\right)=\log\frac{\exp\left(\text{score}\left(X,y{}^{\prime }\right)\right)}{\sum_{i=0}^{n}\exp\left(score\left(X,y\right)\right)}=\text{score}\left(X,y{}^{\prime }\right)-\log\left(\sum_{i=0}^{n}\exp\left(score\left(X,y\right)\right)\right). \end{array}$$

## 3. Data Set and Experimental Evaluation Index

### 3.1. CRF Layer

**Figure 7:**
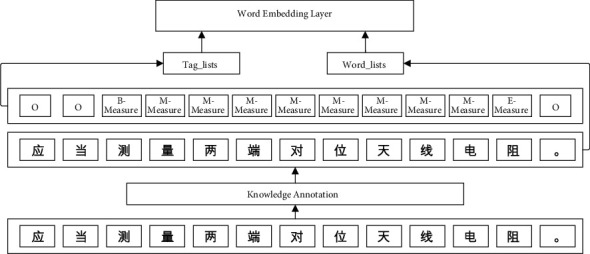
Knowledge annotation and input process of fault text of metro on-board equipment.

The named entity recognition method of subway on-board equipment requires the deep learning method of supervised learning. Therefore, the sample data labeling is required before training. According to the fault knowledge structure, the fault text data of each Metro on-board equipment define three types of named entities: fault location, fault phenomenon, and fault solution. The named entity identification sequence is represented by BMEO, where B (begin) represents the starting position of the entity, *M* (middle) represents the middle part of the entity, and *E* (end) represents the end character of the entity, O (other) represents a nonentity character, and “−” is used to connect the sequence annotation symbol with the defined entity type. Therefore, this paper selects the fault text data recorded in the depot of a subway company from 2016 to 2021 according to the functions and fault characteristics of each equipment. After preprocessing these fault text data, the total amount of data is 51652 marked data, divided into the training set, development set, and test set; 41526 pieces of data are selected as the training set data as the data samples of model fitting; 5035 pieces of data are development set data, which are used to adjust parameters, select features, and make other decisions on learning algorithms; 5091 pieces of data are test set data, used in model evaluation. The knowledge annotation of some data in this paper and the process of input to the model in this paper are illustrated in Figure 7.

### 3.2. Experimental Evaluation Index

In this paper, the Precision, Recall, and *F*_1_ − Score are used as the evaluation indexes of this experiment, whereby TP represents the number of samples classified and divided correctly; FP represents the number of samples classified and divided incorrectly; FN indicates the number of unclassified samples, which are wrong.

#### 3.2.1. Precision

The accuracy rate is only for the positive samples with correct prediction, as opposed to all samples with correct prediction. It is calculated by dividing the number of positive samples with correct prediction by the ratio of the number of positive samples predicted by the model. It shows that the predicted positive samples are really positive, as shown in formula (8):

(18) $$\begin{array}{c} \text{Precision}=\frac{TP}{TP+FP}\times 100\%. \end{array}$$

#### 3.2.2. Recall

It is calculated by dividing the predicted correct number of positive samples by the actual number of positive samples in the test set; it shows that the number of samples that are really positive can be recalled by using the classifier, as shown in formula (9):

(19) $$\begin{array}{c} \text{Recall}=\frac{TP}{TP+FN}\times 100\%. \end{array}$$

#### 3.2.3. F1-Score

*F*_1_ − Score is the harmonic average of accuracy rate and recall rate. Both Precision and Recall are expected to be higher; however, both these indicators are contradictory and cannot both be high. Therefore, *F*_1_ − Score should be introduced as an appropriate threshold point to maximize the ability of the classifier, as shown in formula (10):

(20) $$\begin{array}{c} {\text{F}}_{1}-\text{Score}=\frac{2}{1\text{/Precision}+1\text{/Recall}}\times 100\text{\%}. \end{array}$$

#### 3.2.4. Weighted Average

In this paper, the evaluation indexes Avg − *P*, Avg − *R*, and Avg − *F*_1_ are defined as the weighted average values of 10 entity labels' Precision, Recall, and ,*F*_1_ − Score respectively. The calculation process is shown in formula (21).

(21) $$\begin{array}{c} Avg-P=\frac{\sum_{i=1}^{10}N_{i}\cdot P_{i}}{\sum_{i=1}^{10}N_{i}}Avg-R=\frac{\sum_{i=1}^{10}N_{i}\cdot R_{i}}{\sum_{i=1}^{10}N_{i}}Avg-F_{1}=\frac{\sum_{i=1}^{10}N_{i}\cdot F_{i}}{\sum_{i=1}^{10}N_{i}}. \end{array}$$

Where *i* denotes the value corresponding to the entity category (there are 10 named entity categories in this paper), *N*_*i*_ represents the number of entities in this category, and *P*_*i*_, *R*_*i*_, and *F*_*i*_ represent Precision, Recall, and *F*_1_ − Score corresponding to class *i* entities, respectively.

## 4. Experimental Verification

### 4.1. Experimental Environment

The experimental hardware includes a i7-6700HQ CPU, a GTX960M graphics card, a video memory of 8G, a Win10 64bit operating system, a 3.60 python version, a Spider 5.0.5 development tool, and a 1.11.1GPU Pytorch version.

### 4.2. Experimental Super Parameter Optimization

#### 4.2.1. MHA Attention Heads Number Selection and Other Parameter Settings

The number of attention heads is set to *t* in the MHA layer. During the operation of the MHA layer, the input features need to be divided into *t* parts, and the dimension of the feature vector *X*_*t*_ input to the MHA layer is 320. It is necessary to ensure that the set number of attention heads' *t* value is divisible by 320. Therefore, this paper selects MHA with the number of attention heads of 2, 4, 5, 8, and 10 to test the model in this paper, and the experiment uses all the parameters in Table 1 except attention_heads, and the optimizer chosen is Adam.

**Figure 8:**
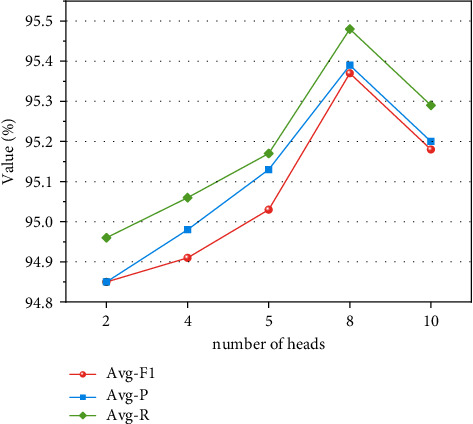
Effects of different attention heads on model performance.

It can be inferred from Figure 8 that after the addition of the multihead self-attention mechanism, the prediction results of the model gradually improves with the increase of the number of self-attention heads; when the head is 8, the Avg − *P*, Avg − *R*, and Avg − *F*_1_ of the model reach the optimum, and the *Avg* − *F*_1_ of the model is increased by 0.52%, 0.46%, and 0.34%, respectively, compared to the number of self-attention heads of 2, 4, and 5. By further increasing the number of heads, the accuracy of the model decreases. This finding arises because an excessive number of attention heads will lead to overfitting of the model. The number of MHA attention heads is set to 8, and the parameter settings are shown in Table 1.

#### 4.2.2. Optimizer Selection

**Figure 9:**
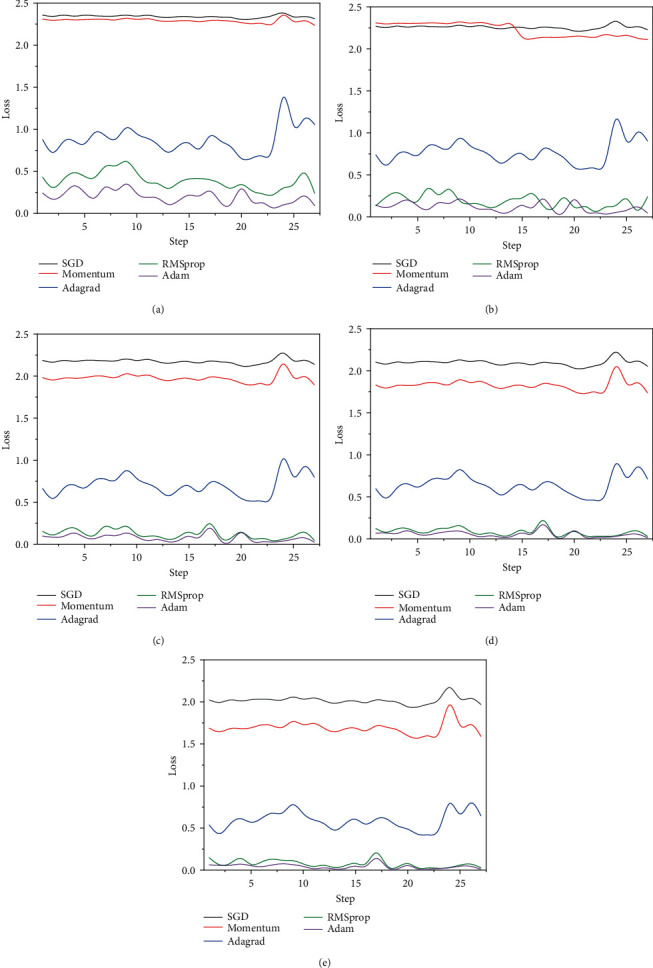
The loss function value of different optimizers varies with the number of iteration steps in different epochs: (a) 5th Epoch; (b) 10th Epoch; (c) 15th Epoch; (d) 20th Epoch; (e) 25th Epoch.

In the deep learning task, the optimizer is used to update and calculate the network parameters affecting the model training and model output, to approximate or reach the optimal value, and to minimize (or maximize) the loss function. In this experiment, five commonly used optimizers are selected: SGD (stochastic gradient descent), momentum optimization method, adaptive learning rate optimization algorithm Adagrad, RMSprop, and Adam. SGD selects a mini batch each time and uses the gradient descent to update the model parameters; Momentum optimization method adds the momentum optimization mechanism based on SGD. The Adagrad algorithm automatically attenuates the learning rate by using the number of iterations and cumulative gradient; RMSprop adds iterative attenuation; The Adam optimizer dynamically adjusts the learning rate of each parameter by using the first-order moment estimation and second-order moment estimation of the gradient [28]. In this experiment, the five optimizers are applied to the named entity recognition training task of this model. The parameter values set in Table 1 are used as the parameter values of this experiment, and The alpha of RMSprop is 0.99 and eps is 1*e* − 08; the beta1 of Adam is 0.9, beta2 is 0.999, and eps is 1*e* − 08, and the rest of the optimizers are the system default parameters. In the process of model training, the variation of loss function value loss with iteration step in the 5th, 10th, 15th, 20th, and 25th epoch rounds is shown in Figures 9(a)to 9(e), respectively.

Adam and RMSprop have the smallest loss function value and stable iterative waveform, while Adam showcases better performance in these two aspects. Therefore, Adam is selected for subsequent experiments in this paper. Moreover, it can be inferred that with the increase of training rounds epoch and training samples in each round, the loss function value loss constantly declines, and finally tends towards stability and 0, indicating that the parameter values in Table 1 optimizes the network training of this model.

### 4.3. Overall Comparison

#### 4.3.1. Experimental Super Parameter Optimization

Several common named entity recognition models in NLP field are used to train the dataset in this paper. After 25 rounds of epoch, the recognition results of 10 entity labels in this paper are generated, as listed in Table 2 respectively. The following comparative analysis is carried out:

The recognition results of phenomenon are shown in Table 2. The *F*1 values of HMM model for BME of phenomenon are 65.52%, 80.33%, and 71.93%, respectively. Compared with (Table 3) CRF model, the F1 value of HMM model for entity (Table 4) label b-phenomenon is 4.81% lower, the F1 value of m-phenomenon is 2.01% lower, and the F1 value of e-phenomenon label is 4.99% lower. These findings arise as the limitation of HMM model is that it uses the trained local model to make a global prediction. The independent BiLSTM model is slightly better than the CRF model for the recognition of these three named entities, and the *F*1 value of Table 5 phenomenon's BME is increased by 2.61%, 2.87%, and 0.97%, respectively, because the single CRF model can only capture the internal relationship between named entities within a certain range. It fails to capture long-distance previous and subsequent information. However, the disadvantage of BiLSTM is that it has a general learning effect on the internal relationship between tags, which can be compensated by combining it with the CRF model. Therefore, the *F*1 value of BiLSTM CRF for entity tag b-phenomenon and e-phenomenon is increased by 0.33% and 1.87%, respectively, compared with BiLSTM, and the *p* value of entity tag m-phenomenon is increased by 3.75%. BiLSTM-CNN-CRF adds a CNN network based on BiLSTM-CRF. The output results of CNN and BiLSTM are fused into the CRF network to complete the named entity work and boost the extraction ability of local features. Therefore, the *F*1 value of BME named entities of phenomenon is increased by 1.4%, 1.63%, and 3.45%, respectively, compared with BiLSTM-CRF, and 1.73%, 0.9%, and 5.23%, respectively, compared with BiLSTM. Moreover, the *P* and *R* values are also improved to varying degrees. BiLSTM-CNN-MHA-CRF adds MHA based on BiLSTM-CNN-CRF to learn the dependency between any two words in the sentence and yields the internal structure information. The *F*1 value of BME three named entities has increased by 0.33%, 1.46%, and 0.83%, respectively. Although the *p* value has decreased slightly, the *R* value has increased by about 4–5 percentage points compared with BiLSTM-CNN-CRF, with a more balanced recognition effect of phenomenon entities.

Most location type named entities correspond to station names, place names, or section names, with relatively fixed names. Therefore, the six models have good recognition effects on location type named entities. The *P*, *R*, and *F*1 of BiLSTM-CNN-MHA-CRF have reached over 90%. Compared with the other five models, the *p* values for BME tag recognition of location are 97.37%, 94.27%, and 94.87%, respectively, which are the highest values. Also, the *F*1 values have reached the highest at 93.67%, 92.79%, and 92.50%, respectively. The recognition effect on the next three BME entity tags is relatively balanced.

The recognition effect of three named entity labels of BME of measure is shown in Table 4. The *F*1 value of b-measure in this paper is only second to BiLSTM-CNN-CRF and BiLSTM-CRF, at 86.73%. The recognition effect of E-MEASURE is general, with *P*, *R*, and *F*1 values of 89.58, 81.69, and 85.45%, respectively. The reason is that the number of these two entity labels is the least, resulting in insufficient learning of these two types of labels in the main model and the inability to play the role in MHA mechanism. However, for the largest number of entity tags with a complex structure, the *P* recognized by the m-measure is 96.23%, which is 4–19 percentage points higher than other models. The *F*1 value reached 91.07%, which is also the highest value among the six models, which is 5–13 percentage points higher than other models.

The recognition effect of other nonentity tags *o* is shown in Table 5. Tag O indicates a type of the label o, as the largest number of 10 tag types, the P, R and F1 values of the proposed six models exceed 94%, while the recognition effect of this model is slightly better. The *P*, *R*, and *F*1 values reach 96.37%, 98.87%, and 97.60%, respectively, and the *F*1 value is 0.6–2 percentage points higher than that of other models.

To sum up, for the identification of the above 10 Tags, the other five models except the model in this paper have relatively good recognition effects on BME entity tag and nonentity tag *o* in location. Also, the model in this paper has better recognition effects on these types of tags. The recognition effect of phenomenon's BME entity label is relatively poor, as the description of fault phenomenon will be detailed to each component. Given the numerous components of subway on-board equipment, the description of fault phenomenon is relatively complex, and the model fitting is more complex. Using the model proposed in this paper, the *F*1 value recognized by BME tag and m-measure tag of phenomenon is substantially improved, and the *F*1 value recognized by b-phenomenon is about 0.4–10 percentage points higher than that of other models. The *F*1 value of m-phenomenon increased by about 1.4–7 percentage points; the *F*1 value of e-phenomenon increased by about 2–4 percentage points; the *F*1 value of m-measure increased by about 5–13 percentage points. Due to the insufficient number of b-measure and E-MEASURE entity labels, the recognition effect of this model on b-measure and E-MEASURE entity labels is general; however, this model improves the recognition effect of 8 entity labels except for b-measure and E-MEASURE.

#### 4.3.2. Overall Recognition Effect

**Figure 10:**
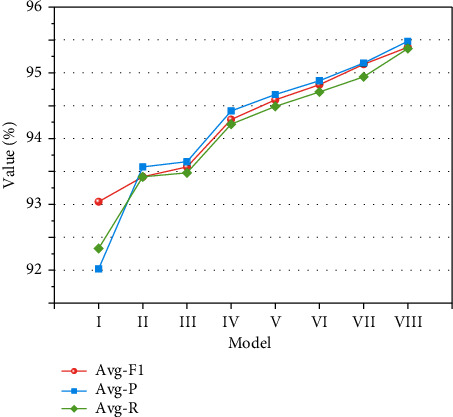
Weighted average of different named entity recognition models.

Further comparison of the weighted average evaluation indexes Avg − *P*, Avg − *R*, and Avg − *F*_1_ of the six models on the recognition results of 10 labels. As depicted in Figure 10 and Table 6, in terms of Avg − *P*, Avg − *R*, and Avg − *F*_1_, the combined model IV–VIII outperforms I, II, and III. Compared with IV, V, and VI, Avg − *P*, Avg − *R*, and Avg − *F*_1_ of V are increased by 0.3%, 0.25%, and 0.27%, respectively, compared with and those of VI is increased by 0.53%, 0.46%, and 0.49%, respectively, compared with IV, indicating that adding CNN can improve the ability of extracting local features. The introduction of MHA largely makes up for the lack of BiLSTM's ability to capture the association relationship between words when processing long sequences and can capture various semantic features and highlight the key information of characters, the level of words and sentences. Therefore, the Avg − *P*, Avg − *R*, and Avg − *F*_1_ of VIII are 0.57%, 0.6%, and 0.66% higher than those of VI, respectively, and the Avg − *P*, Avg − *R*, and Avg − *F*_1_ of VIII are the highest among the six models, 95.39%, 95.48%, and 95.37%, respectively. Both V and VII models connect BiLSTM and CNN in series, whereby the word vector generated by Word2Vec is first input to BiLSTM. The output of BiLSTM is subsequently used as the input of CNN. Compared with V, VI, VII, and VIII, VI has increased by 0.23%, 0.21%, and 0.22%, respectively, in three indexes compared with V; VIII compared with VII, the three indexes are increased by 0.26%, 0.33%, and 0.43%, respectively, indicating that the effect of parallel work of BiLSTM and CNN outperforms serial work.

Therefore, adopting the strategy of BiLSTM and CNN working in parallel and effectively combining MHA and BiLSTM-CNN-CRF can improve the recognition effect compared with other named entity recognition models, which is of great significance to improve the overall performance of the named entity recognition model of Metro on-board equipment.

## 5. Conclusion

Based on unified labeling of Metro on-board equipment fault text data, aimed at solving the problem of low accuracy of naming entity recognition task of unstructured Metro on-board fault data, this paper proposes a Metro on-board equipment naming entity recognition model based on multihead self-attention mechanism and CNN BiLSTM CRF. Compared with the traditional naming entity recognition model BiLSTM CRF, this model adds a CNN network with parallel processing characteristics with BiLSTM. The two extracted features are combined and sent to MHA, which extract the context information and local feature information, and mine the internal relationship between different features through MHA. This paper defines the entity tag BME and other nonentity tags *o* corresponding to the three types, through the named entity recognition experiment of Metro on-board equipment fault text data with this model and other common named entity recognition models, and the experiment results show that:

1. The proposed named entity recognition model has conspicuous advantages in the three indexes of *P*, *R*, and *F*1 for the recognition results of all tags except entity tag b-measure and E-MEASURE, which is higher than HMM, CRF, BiLSTM, BiLSTM-CRF, and BiLSTM-CNN-CRF.
2. The model in this paper has a good performance in the weighted average evaluation indexes Avg − *P*, Avg − *R*, and Avg − *F*_1_, reaching 95.39%, 95.48%, and 95.37%, respectively. It is the highest value when compared with the other five named entity recognition models. Moreover, the strategy of parallel work of BiLSTM and CNN outperforms serial work.

Therefore, it can meet the performance requirements of high accuracy of subway on-board equipment fault text named entity recognition, provide theoretical basis and application value for subway on-board equipment fault named entity recognition, and establish a good foundation for the subsequent establishment of subway on-board knowledge map and the subway on-board knowledge base.

## Figures and Tables

**Table 1:** Parameter setting.

Parameter	Value
Batch_size	64
Learning rate	8*e* − 4
Hidden layer dimension	128
Epoch	25
LSTM_dim	128
cnn_size	64
attention_heads	8
kernel_size	(3, 4, 5)
Activation function	Relu
Max_seq_len	128
Dropout	0.5
Loss function	Cross entropy

**Table 2:** Recognition effect of different named entity recognition models on phenomenon.

Models	B-phenomenon			M-phenomenon			E-phenomenon		
	*P* (%)	*R* (%)	*F*1 (%)	*P* (%)	*R* (%)	*F*1 (%)	*P* (%)	*R* (%)	*F*1 (%)
HMM	55.58	79.17	65.52	74.90	86.61	80.33	62.12	85.42	71.93
CRF	74.42	66.67	70.33	86.49	78.57	82.34	81.40	72.92	76.92
BiLSTM	83.78	64.58	72.94	84.92	85.49	85.21	78.72	77.08	77.89
BiLSTM-CRF	81.25	66.72	73.27	88.67	80.67	84.48	84.85	75.09	79.67
BiLSTM-CNN-CRF	84.85	66.67	74.67	95.59	78.33	86.11	91.43	76.19	83.12
BiLSTM-CNN-MHA-CRF	78.95	71.43	75.00	93.67	82.22	87.57	87.18	80.95	83.95

**Table 3:** Recognition effect of different named entity recognition models on location.

Models	B-location			M-location			E-location		
	*P* (%)	*R* (%)	*F*1 (%)	*P* (%)	*R* (%)	*F*1 (%)	*P* (%)	*R* (%)	*F*1 (%)
HMM	92.92	91.67	92.29	91.26	92.57	91.91	91.18	86.11	88.57
CRF	96.88	86.11	91.18	95.28	86.43	90.64	93.75	83.33	88.24
BiLSTM	96.43	75.00	84.37	92.81	92.14	92.47	91.43	88.89	90.14
BiLSTM-CRF	94.87	90.24	92.50	91.30	90.74	91.02	92.31	87.80	90.00
BiLSTM-CNN-CRF	97.30	87.80	92.31	92.31	87.80	90.00	93.67	91.36	92.50
BiLSTM-CNN-MHA-CRF	97.37	90.24	93.67	94.27	91.36	92.79	94.87	90.24	92.50

**Table 4:** Recognition effect of different named entity recognition models on measure.

Models	B-measure			M-measure			E-measure		
	*P* (%)	*R* (%)	*F*1 (%)	*P* (%)	*R* (%)	*F*1 (%)	*P* (%)	*R* (%)	*F*1 (%)
HMM	67.14	85.42	75.20	77.99	94.88	85.61	75.71	96.36	84.80
CRF	84.62	80.00	82.24	89.40	76.38	82.38	90.38	85.45	87.85
BiLSTM	86.00	78.18	81.90	92.11	68.90	78.83	91.67	80.00	85.44
BiLSTM-CRF	92.59	84.75	88.50	91.37	82.04	86.46	89.29	84.75	86.96
BiLSTM-CNN-CRF	92.41	84.94	88.52	90.76	79.58	84.80	94.34	84.75	89.29
BiLSTM-CNN-MHA-CRF	90.74	83.50	86.73	96.23	86.44	91.07	89.58	81.69	85.45

**Table 5:** Recognition effect of different named entity recognition models on other nonentity tags *O*.

Models	*O*		
	*P* (%)	*R* (%)	*F*1 (%)
HMM	97.70	92.76	95.17
CRF	94.99	97.91	96.43
BiLSTM	95.20	97.63	96.40
BiLSTM-CRF	95.26	98.64	96.92
BiLSTM-CNN-CRF	96.59	98.79	97.01
BiLSTM-CNN-MHA-CRF	96.37	98.87	97.60

**Table 6:** Weighted average of different named entity recognition models.

No	Models	Avg − *P* (%)	Avg − *R* (%)	Avg − *F*_1_ (%)
I	HMM	93.04	92.02	92.33
II	CRF	93.42	93.57	93.42
III	BiLSTM	93.57	93.65	93.48
IV	BiLSTM-CRF	94.29	94.42	94.22
V	(BiLSTM-CNN)_SPI_-CRF	94.59	94.67	94.49
VI	BiLSTM-CNN-CRF	94.82	94.88	94.71
VII	(BiLSTM-CNN)_SPI_-MHA-CRF	95.13	95.15	94.94
VIII	BiLSTM-CNN-MHA-CRF	95.39	95.48	95.37

## Data Availability

The dataset can be obtained from the corresponding author upon request.

## References

[1] Gao C.-H. *A Communication Based on Train Control system*.

[2] Lei J., Tang B., Lu X., Gao K., Jiang M., Xu H. (2014). A comprehensive study of named entity recognition in Chinese clinical text. *Journal of the American Medical Informatics Association*.

[3] Wasserman R. C. (2011). Electronic medical records (EMRs), epidemiology, and epistemology: reflections on EMRs and future pediatric clinical research. *Academic pediatrics*.

[4] Zong C.-Q., Rui X., Zhang J.-J. (2019). *Text Data Mining*.

[5] Pan C.-G. (2012). Research on Chinese named entity recognition based on rule and statistics. *Information Science*.

[6] Zheng J.-H., Li X., Tan H.-Y. (2000). Research on Chinese name recognition method based on corpus. *Chinese Journal of information*.

[7] Bikel D. Nymble: a high-performance learning name-finder.

[8] Bikel D. M., Schwartz R., Weischedel R. M. (1999). An algorithm that learns what’s in a name. *Machine Learning*.

[9] Zhang Y.-J., Xu Z.-T., Xue X.-Y. (2008). Maximum entropy Chinese named entity recognition model based on multi feature fusion. *J], Computer research and development*.

[10] McCallum A., Li W. (2003). Early results for named entity recognition with conditional random fields, feature induction and web—enhanced lexicons [C/proceedings of the seventh conference on natural language learning at HLT — NAACL. Stroudsburg, USA. *ACLPPinforma*.

[11] Ji Z. Y., Kong D. Y., W L. W. D., Sang Y. J. Research on named entity recognition based on deep learning [J/OL]. *Computer integrated manufacturing system*.

[12] Chen K. (2020). *Research on Fault Diagnosis Method of Urban Rail Transit CBTC System Based on Text Mining*.

[13] Liu P., Guo Y.-P., Wang F.-L., Li G.-H. (2022). Chinese Named Entity Recognition: The State of the art. *Neurocomputing*.

[14] Collobert R., Weston J., Bottou L., Karlen L., Kavukcuoglu M., Kuksa P. (2011). Natural language processing (almost) from scratch. *Journal of Machine Learning Research*.

[15] Miao Y. L., Cheng W.-F., Ji Y.-C., Zhang S., Kong Y. L. (2021). Aspect-based sentiment analysis in Chinese based on mobile reviews for BiLSTM-CRF. *Journal of Intelligent and Fuzzy Systems*.

[16] Chen X., Qiu X., Zhu C., Liu P., Huang X. J. Long short-term memory neural networks for Chinese word segmentation.

[17] Zhou Q.-H., Li X.-L. Research on fault short text classification method of railway signal equipment based on MCNN. *Journal of Railway Science and Engineering*.

[18] Ye N., Qin X., Dong L., Zhang X., Sun K. (2020). Chinese named entity recognition based on character-word vector fusion. *Wireless Communications and Mobile Computing*.

[19] Kim Y. (2014). *Convolutional Neural Networks for Sentence Classification*.

[20] Gao J., Zhang Z.-P., Cao P., Huang W., Li F.-F. (2021). Citation entity recognition method using multi‐feature semantic fusion based on deep learning. *Concurrency and Computation: Practice and Experience*.

[21] Ma X., Hovy E. End-to-end Sequence Labeling via Bi-directional LSTM-CNNs-CRF.

[22] Ayifu M., Wushouer S., Palidan M. (2019). Multilingual named entity recognition based on the BiGRU-CNN-CRF hybrid model. *International Journal of Information and Communication Technology*.

[23] Liu X.-L., Zhang M.-Q., Gu Q., Ren Y.-Z., He D.-B., Gao W.-L. (2021). Named entity recognition of fresh egg supply chain based on BERT-CRF model. *Journal of agricultural machinery*.

[24] Yang L.-B. (2018). *Research and Application of Key Technologies of Railway Accident Fault Text Big Data Analysis*.

[25] Yang J., Zhang Y., Li L., Li X. YEDDA: A Lightweight Collaborative Text Span Annotation Tool.

[26] Dheeraj K., Ramakrishnudu T. (2021). Negative emotions detection on online mental-health related patients texts using the deep learning with MHA-BCNN model. *Expert Systems with Applications*.

[27] Li C.-F., Ma K. (2022). Entity recognition of Chinese medical text based on multi-head self-attention combined with BILSTM-CRF. *Mathematical Biosciences and Engineering*.

[28] Adewumi T. P., Liwicki F., Liwicki M. (2020). Word2Vec: Optimal Hyper-Parameters and Their Impact on NLP Downstream Tasks. *Open Computer Scienc*.

[29] Graves A. (2012). *Supervised Sequence Labelling*.

[30] Sun R. (2019). Optimization for Deep Learning: Theory and algorithms. https://arxiv.org/abs/1912.08957.

